# Nutritional Epigenomics and Age-Related Disease

**DOI:** 10.1093/cdn/nzaa097

**Published:** 2020-06-06

**Authors:** Sophia D Amenyah, Mary Ward, J J Strain, Helene McNulty, Catherine F Hughes, Caitlin Dollin, Colum P Walsh, Diane J Lees-Murdock

**Affiliations:** Genomic Medicine Research Group , School of Biomedical Sciences, Ulster University, Northern Ireland, United Kingdom. BT52 1SA; Nutrition Innovation Centre for Food and Health (NICHE), School of Biomedical Sciences, Ulster University, Coleraine, Northern Ireland, United Kingdom. BT52 1SA; Nutrition Innovation Centre for Food and Health (NICHE), School of Biomedical Sciences, Ulster University, Coleraine, Northern Ireland, United Kingdom. BT52 1SA; Nutrition Innovation Centre for Food and Health (NICHE), School of Biomedical Sciences, Ulster University, Coleraine, Northern Ireland, United Kingdom. BT52 1SA; Nutrition Innovation Centre for Food and Health (NICHE), School of Biomedical Sciences, Ulster University, Coleraine, Northern Ireland, United Kingdom. BT52 1SA; Nutrition Innovation Centre for Food and Health (NICHE), School of Biomedical Sciences, Ulster University, Coleraine, Northern Ireland, United Kingdom. BT52 1SA; Genomic Medicine Research Group , School of Biomedical Sciences, Ulster University, Northern Ireland, United Kingdom. BT52 1SA; Genomic Medicine Research Group , School of Biomedical Sciences, Ulster University, Northern Ireland, United Kingdom. BT52 1SA; Genomic Medicine Research Group , School of Biomedical Sciences, Ulster University, Northern Ireland, United Kingdom. BT52 1SA

**Keywords:** aging, B-vitamins, diet, DNA methylation, epigenetic age, epigenetic age acceleration, epigenetic clock, one-carbon metabolism

## Abstract

Recent advances in epigenetic research have enabled the development of epigenetic clocks, which have greatly enhanced our ability to investigate molecular processes that contribute to aging and age-related disease. These biomarkers offer the potential to measure the effect of environmental exposures linked to dynamic changes in DNA methylation, including nutrients, as factors in age-related disease. They also offer a compelling insight into how imbalances in the supply of nutrients, particularly B-vitamins, or polymorphisms in regulatory enzymes involved in 1-carbon metabolism, the key pathway that supplies methyl groups for epigenetic reactions, may influence epigenetic age and interindividual disease susceptibility. Evidence from recent studies is critically reviewed, focusing on the significant contribution of the epigenetic clock to nutritional epigenomics and its impact on health outcomes and age-related disease. Further longitudinal studies and randomized nutritional interventions are required to advance the field.

## Introduction

Epigenetic regulation has been identified as a key factor in aging (1) and is linked with diet, metabolism, and disease (2, 3). During the last decade, novel epigenetic clock models to identify DNA methylation signatures that accurately predict chronological age, disease, and mortality, have also provided a measure of epigenetic or biological age. Epigenetic clocks offer immense potential to improve our understanding of the significant current global challenge of the disparity between the lengthening of average lifespan (4), which has not been matched by similar improvements in healthspan, with relatively static rates of age-related disease (5). During the last decade, the application of epigenetic clock models to data generated by epigenome-wide association studies (EWAS) focused on dietary intakes and nutritional intervention is helping to uncover dietary determinants of healthy aging.

Maintaining optimal nutritional status will have an important contribution to improving health outcomes with respect to age-related disease and healthspan. Several dietary factors are emerging as key modifiers of biological age and epigenetic clock models are helping to unravel the complex interplay of diet and age-related disease. Folate and related B-vitamins, essential cofactors in 1-carbon metabolism, the main metabolic pathway for generating methyl groups for DNA methylation (6), are emerging as factors that can modify epigenetic age. Perturbations to DNA methylation owing to imbalances in the supply of B-vitamins, or to polymorphisms or interactions between the various regulating enzymes, could lead to aberrant DNA methylation and subsequently influence epigenetic age and disease susceptibility (7).

Suboptimal B-vitamin status is associated with accelerated aging of the brain, declining cognitive function, and cardiovascular disease (CVD), indicating that B-vitamins may play protective roles in age-related disease (8–10). High prevalence of low dietary intakes for B-vitamins (i.e., below the estimated average requirement; EAR), including folate (29–35%), vitamin B-6 (24–31%), and riboflavin (31–41%) have been reported in older adults (11). More recent estimates from older adults (*n* = 5290; ≥50 y) from the Irish Longitudinal Study on Ageing (TILDA) (Wave 1) and (*n* = 5186) from the Trinity Ulster Department of Agriculture (TUDA) study reported the prevalence of deficient or low vitamin B-12 status (<185 pmol/L) as 12% and 11.8%, respectively, whereas the prevalence of deficient/low folate status was ≤15% (12, 13).

Application of epigenetic clock models to epigenomic data from dietary interventions or longitudinal studies of dietary intake offer immense potential for elucidating how nutrition can modulate age-related disease processes and improve health outcomes. As the volume of studies investigating the effect of nutrients, in particular B-vitamins, on DNA methylation in health and disease begin to increase, understanding the essential role of these nutrients in modulating DNA methylation age and age acceleration are critical.

The aim of this literature review was to address this gap by providing a critical overview of recent studies using the epigenetic clock to predict biological age and age-related disease and the application of nutrition in modifying these parameters. Further longitudinal studies and randomized nutritional interventions are required. Additionally, challenges with methodology are highlighted and opportunities presented for researchers to consider for advancement of the field of nutritional epigenomics and age-related disease.

### Literature search strategy

The literature search for this review was conducted by searching the Medline (via OvidSP) database and PubMed for articles published in English only and limited to human studies. Both medical subject headings and keywords were used in the search to identify articles with relevant information on aging, DNA methylation clock, diet, and vitamins. This was subsequently followed by forward citation searching or “snowballing” whereby relevant references were identified from key articles, followed up, and repeating the process with each article used to obtain more literature.

Medical subject headlines included: exp DNA Methylation/, exp Dietary Supplements/, exp Micronutrients/, Vitamins/, Vitamin B Complex/, Food, Fortified/, genome-wide methylation.mp. or Methylation/, Aging/or Biological Clocks/or Epigenetic clock.mp. or DNA Methylation/. The keywords used were: (diet or nutrient or cobalamin or folate or methionine or betaine or choline or riboflavin or “vitamin b2” or “vitamin b12”) or (“methylation clock” or 450K or Methyl450 or Methylation450 or beadchip or “bead chip” or 800k or epic or EWAS or genome-wide or genomewide or epigenome-wide or epigenomewide). Finally, only those articles with emphasis on vitamins, diet, micronutrients, and methylation clocks were selected, and the relevant data was extracted for the review.

## DNA Methylation and 1-Carbon Metabolism

### DNA methylation

**FIGURE 1:**
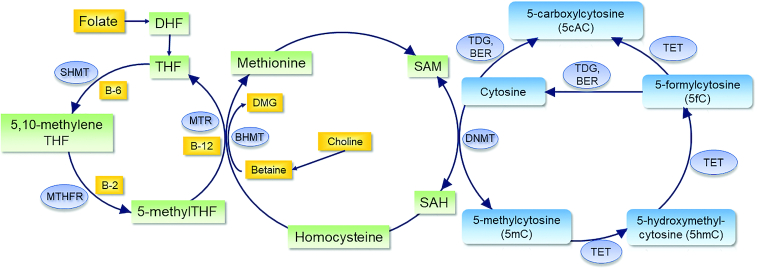
Brief summary of 1-carbon metabolism and DNA methylation. BER, base excision repair enzymes; BHMT, betaine-homocysteine S-methyltransferase; DHF, dihydrofolate; DMG, dimethylglycine; DNMT, DNA methyltransferase; MTHFR, methylenetetrahydrofolate reductase; MTR, methionine synthase; SAH, S-adenosylhomocysteine; SAM, S-adenosylmethionine; SHMT, serine hydroxymethyltransferase; TDG, thymine DNA glycosylase; TET, ten-eleven translocation methylcytosine dioxygenase enzymes; THF, tetrahydrofolate.

DNA methylation is widely regarded as the most stable epigenetic mark involved in establishing patterns of gene expression and phenotype (14). It usually involves the covalent binding of methyl groups to the 5’ position of a cytosine (5C) to form 5’-methylcytosine (5mC) and occurs within CpG dinucleotide sequences (15). DNA methylation may also occur at non-CpG sites, such as CpA, CpT, and CpC; however, the functions and mechanisms of such methylation and implications for gene expression are currently not fully understood (16). This review, therefore, focuses on DNA methylation at 5mC. Methylation reactions are catalyzed by a family of DNA methyltransferases (DNMTs) which transfer a methyl group from S-adenosylmethionine (SAM) (**Figure 1**). Removal of DNA methylation can occur via passive (failure to maintain methylation following replication) or active mechanisms. Active demethylation is carried out by the ten-eleven translocation methylcytosine dioxygenase enzymes (TETs) recently reviewed in (17) to produce 5mC derivatives, 5’-hydroxymethylcytosine (5hmC), 5’-formylcytosine (5fC), and 5’-carboxylcytosine (5caC). Additionally, the 5caC is then removed through the action of the base excision repair enzyme thymine DNA glycosylase (TDG) (18) (Figure 1).

DNA methylation has differing functions, depending on its location within the genome. It is usually associated with transcriptional gene repression at CpG-rich promoters; however, a mechanistic link between gene body methylation and active transcription is also suggested by enrichment of 5mC within gene bodies of transcribed genes (19). CpG sites dispersed throughout the genome are usually methylated (20, 21), unlike CpGs lying within distinct, CG-rich CpG islands (CGIs), often found in the promoters of housekeeping genes (22), which are mostly unmethylated (20, 21). In the regions immediately neighboring CpG islands, CpG shores (≤2 kb from CGI) and CpG shelves (2–4 kb from CGI), display higher levels of methylation, with variations at these locations having a stronger impact on gene expression than the CpG island and may account for tissue-specific expression and disease variability (23, 24). Additionally, methylation occurring at other genomic regions including transcription start sites (TSS) and intergenic regions has also been shown to influence transcription and gene expression (25, 26).

DNA methylation modifications are dynamic, extensively reprogrammed in early development (27, 28), and continue to a lesser, but nonetheless important extent throughout the lifespan, owing to the influence of various environmental conditions, particularly diet, which importantly contribute to both the aging process and disease susceptibility (29).

### Influence of nutrients on DNA methylation

One-carbon metabolism provides a direct link between nutrients, mainly folate and related B-vitamins, and DNA methylation (Figure 1) and therefore has become of interest to investigate in epigenetic studies. The interconnected biochemical pathways generate methyl groups for the synthesis of purines and thymidine, and biological methylation reactions including DNA, RNA, and histone methylation. Folate and related B-vitamins: vitamin B-12, vitamin B-6, and the largely overlooked vitamin B-2 (riboflavin), and other nutrients including methionine, choline, and betaine provide substrates and cofactors to help the efficient functioning of the system. Folate from the diet or in the synthetic form, folic acid, is converted to 5-methyltetrahydrofolate (5-mTHF) and dihydrofolate (DHF), respectively, and subsequently to tetrahydrofolate (THF) (30). Tetrahydrofolate is then converted to 5,10-methylenetetrahydrofolate and subsequently to 5-mTHF by methylenetetrahydrofolate reductase (MTHFR) with vitamin B-2 (riboflavin) as a cofactor. 5-mTHF is then demethylated as the 1-carbon is donated for remethylation of homocysteine to methionine by methionine synthase (MTR) with vitamin B-12 as a cofactor (31). 5,10-methylenetetrahydrofolate dehydrogenase (MTHFD1), catalyzes the conversion of tetrahydrofolate to 10-formyl, 5,10-methenyl, and 5,10-methylene derivatives subsequently used as cofactors for de novo purine and pyrimidine synthesis (30, 32). The choline-betaine pathway is a parallel pathway that involves the transfer of a methyl group from betaine to homocysteine, a vitamin B-6 dependent reaction, to produce dimethylglycine (DMG) and methionine.

Methionine regenerated from homocysteine serves as a precursor for SAM and is then converted to S-adenosylhomocysteine (SAH) during the methyl transfer (33). The cellular potential for DNA methylation relies upon the relative amounts of the methyl donor SAM and its reaction product SAH (34). The effects of dietary intake or supplementation with B-vitamins has been shown in a limited number of studies to increase SAM concentrations (35, 36). Supplementation with riboflavin (1.6 mg/d for 16 wk) and folic acid (5 mg/d for 8 wk) increased mean plasma SAM concentrations in adults with the *MTHFR* 677TT genotype (35, 36). It has been postulated that the higher the SAM:SAH ratio, the greater the methylation potential of the cell, although conflicting evidence suggests that DNA methylation may proceed without changes in the ratio (37, 38). Further studies are required to clarify the effect of dietary molecules on SAM concentrations and DNA methylation.

Perturbations in 1-carbon metabolism may occur through low intake of nutrients involved in 1-carbon metabolism (7), malabsorption of nutrients via disease or cellular conditions, interactions in regulatory enzymes in 1-carbon metabolism pathways as well as common polymorphisms within genes that code for enzymes important for the normal functioning of 1-carbon metabolism (2, 39). Apart from significant disruption to 1-carbon metabolism, these perturbations may have functional implications on downstream biological processes including DNA methylation and synthesis.

### Common polymorphisms in genes involved in 1-carbon metabolism

Common polymorphisms in genes involved in 1-carbon metabolism can influence enzyme activities, and subsequently metabolite and substrate concentrations in the pathway. The *MTHFR* C677T polymorphism results in reduced MTHFR enzyme activity in individuals with the 677TT genotype which encodes a thermolabile enzyme (40). Elevated plasma homocysteine indicates perturbed 1-carbon metabolism in 677TT individuals, and it is plausible that altered concentrations of SAM and, therefore, availability of methyl donors for methylation reactions may ensue. The well-established phenotype of elevated homocysteine is widely reported in different populations. A large-scale population-based study (*n* = 10,601) found strong associations of *MTHFR* c665C > T polymorphism with blood concentrations of total plasma homocysteine and serum folate (41). The 665TT genotype was associated with a higher concentration of homocysteine and lower concentration of folate than the 665CC genotype, with the CT genotype having intermediate concentrations. Riboflavin supplementation in a randomized controlled trial (RCT) of adults reduced plasma homocysteine specifically in 677TT individuals (42) indicating that riboflavin may stabilize the thermolabile enzyme and restore MTHFR activity, and thus is a very interesting nutrient for future epigenetic investigations. A recent study by our group using evidence from RCTs showed that supplementation with riboflavin resulted in decreased global and *MTHFR* north shore methylation in adults with the *MTHFR* 677TT genotype (43).

Polymorphisms can also act as strong *cis*-regulatory elements (*cis*-meQTL; *cis*-methylation quantitative trait loci) to regulate the methylation levels of their own gene promoter or *trans*-regulatory elements (*trans*-meQTL) regulating methylation of other genes. For example, 57 CpGs were differentially methylated depending on the genotype of 6 1-carbon metabolism genes (*FTHFD, MTHFD_1_, MTHFR, MTR, MTRR*, and *TYMS; P* <0.5 × 10^−5^). The *MTHFR* rs1801133 single nucleotide polymorphisms (SNPs) (responsible for the C677T polymorphism) was shown to act as a *trans*-meQTL regulatory element in breast tissue associated with lower methylation of 5 CpGs (*CLEC17A, DLX6AS*, cg13811423, cg14118666, and cg181152144; average OR = 0.15; average 95% CI: 0.05–0.42) (44). The *MTHFR* promoter itself is also a target for *trans*-meQTL regulatory elements such as the *DNMT3B* −149C > T polymorphism. Increasing the number of T alleles at this position significantly increased *MTHFR* methylation with the *DNMT3B* −149CC genotype having significantly lower levels of *MTHFR* methylation than the CT genotype, which in turn had significantly lower levels of methylation than subjects with the TT genotype (45).

### Role of DNA methylation and diet in aging and disease

The aging process is complex and involves numerous changes at both the molecular and cellular level, including epigenetic remodeling of the DNA methylome (46, 47). DNA methylation patterns, established early in development, progressively diverge throughout the life course, with age-associated DNA methylation features identified by middle-age at a large number of CpG sites continuing to undergo changes into old age (48). Changes in DNA methylation associated with age have been observed in many cross-sectional studies; however, longitudinal evidence which is not confounded by interindividual differences is more limited. In such studies, longitudinal analysis of a cohort of elderly twin pairs identified 2284 CpG sites where DNA methylation levels changed over a 10-y follow-up period (49). A 20-y study of 385 older Swedish twins also identified 1316 longitudinal age-associated methylation sites that were validated in 2 independent cohorts (50). Although it is now well accepted that epigenetic alterations are hallmarks of aging, understanding the causality between these epigenetic changes and the aging process has not been fully elucidated and is still an active area of investigation (51). Multiple studies have reported not only significant associations between aging and DNA methylation (52, 53) but also associations between age-related diseases and epigenetic alterations. The processes that drive the changes in the aging methylome, and subsequent implications for disease and mortality risk are currently not well understood; however, several potential mechanisms have been proposed. These include effects on immunity and inflammation, whereas environmental factors, such as diet, stress, physical activity, socioeconomic status, and smoking (52, 54, 55, 56) could impact these mechanisms or act directly to age the methylome. Aging-associated immune-system impairments are mediated via changes in DNA methylation in nonagenarians. In a cross‐sectional analysis of 4173 postmenopausal females, age-related changes in immune functioning and inflammation were also shown to contribute to increased susceptibility to a wide range of diseases (57, 58).

Dietary factors, particularly B-vitamins, may modulate DNA methylation and thereby influence age-related disease. In studies investigating B-vitamins and DNA methylation in disease, Fiorito and colleagues (59) reported that DNA methylation of specific genes (*TCN2*, *CBS*, *PON1*, *AMT*) involved in 1-carbon metabolism and homocysteine metabolic pathways could mediate the CVD risk conferred by low dietary intake of B-vitamins. Furthermore, using highly robust and comprehensive microarray methods, several large EWAS have shown that supplementation with B-vitamins, predominantly folate and vitamin B-12, or dietary intake of these nutrients modulate DNA methylation at the genome-wide level in older adults (**Table 1**), highlighting key targets that could be further explored in age-related nutritional epigenomics studies (60, 61). Riboflavin has not been as widely studied as other B-vitamins with only 1 EWAS reporting the effects of variability in dietary intake on DNA methylation. Low dietary intake of riboflavin was associated with higher methylation at 1 CpG (*cg21230392; P* = 5×10-8) in a study involving participants from the Melbourne Collaborative Cohort Study (MCCS) (62). Additionally, supplementation with flavanols and polyphenols may affect the activity of enzymes including DNMTs and significantly impact methylation (63). For example, (-)-epigallocatechin-3-gallate (EGCG), a key polyphenol in tea inhibits DNMT activity resulting in demethylation and reactivation of methylation-silenced genes in cancer cells. Further evidence from RCTs of nutrients, such as riboflavin supplementation could elucidate how individual nutrients influence the epigenome and age-related disease.

## Epigenetic Clocks

### Epigenetic drift versus epigenetic clock

**FIGURE 2:**
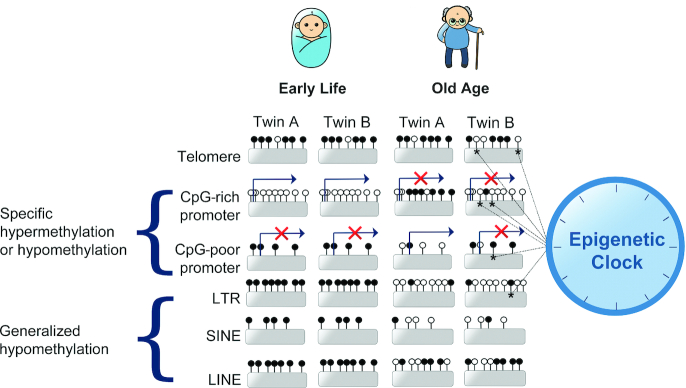
DNA methylation patterns of monozygotic twins diverge during aging. Despite early similarities, stochastic changes occur in the methylome of each twin, A and B during aging. Epigenetic drift results in age-related hypermethylation of CpG-rich sequences such as CGI promoters, typically found in ubiquitously expressed housekeeping genes, which may be switched off as a result of aberrant age-related methylation. In contrast, highly methylated, transcriptionally repressed CpG-poor promoters tend to become hypomethylated during aging, leading to aberrant gene expression. Tandem satellite repeat sequences in the telomere are also heavily methylated which may promote genome stability and inhibit recombination. Hypermethylated interspersed repeats such as LTRs, SINEs, and LINEs tend to undergo generalized hypomethylation during aging. ^⋆^A selection of CpGs undergo programed reproducible methylation changes across the population during aging and have been incorporated into epigenetic clock algorithms used to accurately predict epigenetic age. Each lollipop represents an individual CpG, arrows indicate transcription start sites, X indicates transcriptional repression. CGI, CpG island; LINE, long interspersed nuclear element; LTR, long terminal repeat; SINE, short interspersed nuclear element.

Studies of monozygotic (MZ) twins have shown that although twins are epigenetically indistinguishable during the early years of life, older MZ twins exhibited remarkable differences in their epigenome, indicating that patterns of epigenetic modifications in MZ twin pairs diverge as they become older (64). Entropic decay of DNA methylation during aging is observed with twin studies also revealing that repeat sequences generally become more hypomethylated during aging (65, 66), with methylation increases noted at individual regulatory locus-specific regions (67) (**Figure 2**). Tissue-dependent DNA methylation variation may explain why particular organs and tissue are susceptible to different diseases (68). Many methylation changes leading to interindividual divergence occur stochastically during aging and are known as “epigenetic drift.” Specific CpG sites have been identified to undergo reproducible methylation changes across individuals with age allowing their utilization in epigenetic clock algorithms (69), which can be used to accurately predict chronological age and estimate biological age (Figure 2).

### Epigenetic clocks and age acceleration

Chronological age as a predictor of disease risk and mortality is suboptimal as individuals with the same chronological age may exhibit different susceptibility to age-related diseases owing to differences in underlying biological aging processes (70). This has led to the advent of several DNA methylation-based models of biological aging known as epigenetic clocks **(****Table 2****)**. Each clock is derived by a linear regression algorithm that trains against the chronological age of sample donors and selects a set of CpGs, determining the weighted contribution of each CpG in the set to produce a DNA methylation age (DNAm Age) that correlates accurately with chronological age. The first of these to have a major impact was the Horvath clock (69), which analyses methylation at 353 CpGs and was developed using a panel of 51 different noncancerous tissues and cell lines, leading to it being known as a pan-tissue clock. This feature has enabled accurate predictions of DNAm Age across heterogeneous tissues and cell types. Owing to the wide age range of individuals from which the samples were derived, the Horvath clock is also known as a life course clock and is applicable to analysis of epigenetic age in children and perinatal samples (71). The Hannum methylation clock (56) was derived from the analysis of whole blood in 482 individuals of either Caucasian or Hispanic ethnicity using 71 CpGs to provide superior accuracy in age determination. A recent meta-analysis of over 41,607 participants indicated that each 5-y increase in DNA methylation age, estimated using either the Horvath or Hannum clocks, was associated with an 8–15% increased risk of mortality (72).

**TABLE 1:** Dietary influence on DNA methylation using the Illumina microarray platforms

Study	Study design	Population	Sample size (*n*)	Dietary factor	Source of DNA	Effect
Randomized controlled trials and intervention studies						
Kok et al., 2015 (73)	RCT	B-vitamins for the Prevention of Osteoporotic Fractures (B-PROOF) study	87	Folic acid, vitamin B-12 supplementation	Buffy coat	Differential methylation at 162 positions upon FA/vB-12 supplementation (1 DMP, cg19380919 sig) in intervention compared with placebo
						6 DMRs differed significantly between intervention and placebo groups. Serum folate and vitamin B-12 significantly related to DNA methylation of 173 and 425 regions, respectively
Arpon et al., 2016 (74)	Intervention study	PREDIMED study	36	Mediterranean diet supplemented with extra virgin olive oil	Peripheral blood cells	Med Diet is associated with differential methylation of inflammation-related genes
Cross-sectional studies						
Chamberlainet al., 2018 (62)	Cross-sectional	Melbourne Collaborative Cohort Study (MCCS)	5186	Dietary intake of folate, riboflavin, vitamins B-6 and B-12, methionine, choline, betaine	Peripheral blood	Low intake of riboflavin associated with higher methylation at CpG cg21230392 (*P* = 5 x 10^-8^)
Mandaviya et al., 2019 (75)	Cross-sectional	10 cohorts from Europe and the USA	5841	Dietary intake of folate, vitamin B-12	Leukocytes	6 DMPs and 73 DMRs negatively associated with folate intake. Intake of vitamin B-12 associated with 29 DMRs
Perrier et al., 2019 (76)	Cross-sectional	The European Prospective Investigation into Cancer & Nutrition(EPIC) study	450	Dietary intake of folate	Buffy coat	Dietary intake of folate associated with differential methylation at 24 regions (FDR, *P* < 0.05)

DMP, differentially methylated position; DMR, differentially methylated region; FA, folic acid; FDR, false discovery rate; PREDIMED, Prevención con Dieta Mediterránea; RCT, randomized controlled trial ; vB, vitamin B12 .

When biological age (DNAm Age) exceeds chronological age, age acceleration (AgeAccel) is said to be experienced and this measure is perhaps of most interest to scientists and clinicians studying aging and disease. AgeAccel is defined as the residual from regressing DNAm Age on chronological age, where a positive value indicates that epigenetic age is greater than expected. Horvath further characterized epigenetic age acceleration as either intrinsic (IEAA) or extrinsic (EEAA) epigenetic age acceleration. IEAA is a measure of age acceleration that is independent of age-related changes in the cellular composition of blood, whereas EEAA captures the age-related functional decline of the immune system and accounts for changes in blood cell composition such as the decrease of naïve CD8+ T cells and the increase in memory or exhausted CD8+ T cells (77).

**TABLE 2:** Key features of epigenetic DNA methylation clocks

DNA methylation clock	Number of CpGs	Platform used in development	Tissues used in training	Training set	Key features
Horvath (69)	353	27K & 450K	Multiple tissues (*n* = 51)	Multiple studies, *n* = 7844, mean age 43 y	Predicts methylation age across the lifespan
					Can be applied to children and prenatal samples
					Provides estimates of both intrinsic and extrinsic epigenetic age
					Estimations may be biased in older adults
Hannum (56)	71	27K & 450K	Blood	2 cohorts, *n* = 656 (*n*_1_ = 482; *n*_2_ = 174), age range 19–101 y	Tailored to adult blood samples and may lead to biased estimates in children and in nonblood tissues
					Age estimations may be confounded by age-related changes in blood composition
					Provides a more accurate prediction of life expectancy than Horvath clock
PhenoAge (78)	513	27K, 450K, & EPIC	Blood	2-step process: *1*) phenotypic age; NHANES-III, *n* = 9926, age >20 y	Biomarker relates to numerous age-related diseases and disease phenotypes
				*2*) Epigenetic marker of phenotypic age; InCHIANTI, *n* = 456, age range 21–100 y	Improved predictive power over previous Horvath & Hannum clocks
					Incorporates 9 age-related biochemical measures and smoking-related changes in DNA methylation
					Captures organismal age and the functional state of organs and tissues
					Estimations may be biased in children and in nonblood tissues
GrimAge (79)	1030	450K & EPIC	Blood	Framingham Heart Study (FHS), *n* = 2536 divided into:	DNA methylation surrogates developed for 7 plasma proteins plus smoking pack years
				*1*) training set *n* = 1731 from 622 pedigrees, mean age 66 y	Currently best predictive epigenetic biomarker for lifespan and time to coronary heart disease (18% and 61%, respectively), more predictive than chronological age
				*2*) test set *n* = 625 from 266 pedigrees, mean age 67 y	Highlights healthy diet and educational attainment as predictors of biological age

Summary of the key features of the 4 current epigenetic clocks, including the number of CpGs included in algorithm, the platforms and tissues used in development and the tissues used in training. 27K, Infinium 27K BeadChip array; 450K, HumanMethylation450K BeadChip array; EPIC, Infinium Methylation EPIC BeadChip (850K) microarray; InCHIANTI, Invecchiare in Chianti, aging in the Chianti area.

To investigate biological age more extensively and discriminate morbidity and mortality more accurately among individuals of the same chronological age, recently developed clocks have been trained on age-related and disease phenotypes in combination with chronological age. Two of the most robust are the DNAm Phenotypic Age predictor (DNAm PhenoAge) (78) and the DNAm-based biomarker of mortality GrimAge (DNAm GrimAge) (79). The PhenoAge clock calculates phenotypic age in a 2-step process. Initially, 42 clinical blood biomarkers that predict mortality in NHANES III were used to derive an estimate of phenotypic age. Subsequently, refinement to select 9 of these biomarkers plus chronological age were used independently of DNA methylation to predict phenotypic age. In the final model, a phenotypic age was calculated in the independent Invecchiare in Chianti (InCHIANTI) cohort and a DNA methylation proxy of phenotypic age (DNAm PhenoAge) and age acceleration (AgeAccelPheno) were derived based on a set of 513 CpGs. The Horvath and Hannum clocks are not influenced by smoking status; however, the DNAm PhenoAge clock includes this disease-related factor associated with DNA methylation changes. The PhenoAge clock was found to outperform the Horvath and Hannum epigenetic age measures with respect to a variety of aging outcomes, including all-cause mortality, cancers, healthspan, physical functioning, and Alzheimer's disease (78). The most recent of these biological clocks, DNAm GrimAge, was trained using the Framingham Heart Study (78) and tracks methylation of CpGs of blood-based protein biomarkers that are known to be associated with health such as plasminogen activation inhibitor 1 (PAI-1) and growth differentiation factor 15 (GDF15), as well as a more sensitive measure of CpGs associated with smoking through an estimate of “pack years.” Incorporation of valuable information from these loci has resulted in improvements in accuracy of age acceleration (GrimAgeAccel), which has been shown to be 18% more accurate than chronological age and 14% more accurate than previously described clocks in predictions of time to disease (42). DNA methylation age is currently one of the most accurate measures of aging and life expectancy in a range of traditional measures such as telomere length, and proteomic, transcriptomic, and metabolomic biomarkers in accurately estimating biological age (80).

The CpGs which are included in the clock algorithms are widely distributed across the genome and do not appear to be clustered in or near any particular genomic feature or any particular regulatory region. The methylation clocks and associated challenges have been extensively reviewed recently (81, 82). It is important to note that, although these clocks are highly correlated with chronological age, they were constructed using different algorithms which may influence their prediction of disease and health outcomes; therefore, careful consideration should be given to the most appropriate clock to utilize in any given study.

Epigenetic clocks are not linear across the lifespan. Many of the current epigenetic clock studies have been conducted in adults, and as a result, many show impressive accuracy across most tissues during middle age (83). In later life, however, chronological age increases at a faster rate than epigenetic age, particularly in the Horvath and Hannum clocks (84). A nonlinear pattern is also observed in the clock during childhood (71) and teenage years, due to a greater rate of DNA methylation change in children than adults (85). The Horvath clock has been adjusted to include a log linear transformation for data points from younger individuals and a new clock trained on pediatric buccal swabs has increased predictive power in samples from children (86). Furthermore, as none of the clocks are well-suited to estimating gestational age, the recent development of a placenta clock can be used to closely track fetal age during development (87).

### Epigenetic age, age acceleration, and health outcomes

Epigenetic age and age acceleration are strongly linked to all-cause mortality, higher cancer and CVD mortality and are associated with important inflammatory biomarkers including C-reactive protein, IL6, and monocyte chemotactic protein (88, 89). **Table 3** provides an overview of age-related conditions, DNA methylation age, and age acceleration measured by the 4 different clocks. Although the list is not comprehensive, it is indicative of the broad range of age-related diseases associated with altered epigenetic age. Of particular note, CVD and related measures such as blood pressure have emerged as age-related conditions that are robustly correlated with methylation in a range of epigenetic clocks. Accelerated PhenoAge is associated with a higher risk of coronary heart disease (β = 0.016–0.073; Meta *P* = 3.35 x 10^-11^) and both higher EEAA (r = 0.07, *P* = 4 x 10^-6^) and AgeAccelPheno (r = 0.08, *P*  = 1 x 10^-6^) are associated with elevated systolic blood pressure (58, 78). GrimAgeAccel also gives the most accurate predictions of time-to-coronary heart disease (HR = 1.07, *P* = 6.2 x 10^-24^) and time-to-cancer (HR = 1.07, *P* = 1.3 x 10^-12^) and also demonstrates a strong association with hypertension (OR = 1.04, *P* = 5.1 x 10^-13^) (79).

### Epigenetic age, age acceleration, and dietary factors

The influence of diet in the etiology of many age-related diseases is well established and the advent of epigenetic clocks has brought a novel approach to confirm diet as an important health factor (79). Epigenetic age and age acceleration are linked to a variety of dietary factors such as fish, fruit, and vegetable intakes indicating that a healthy diet and lifestyle could positively influence epigenetic age acceleration (**Table 4**). For example, a previous study highlighted that ω-3 PUFA supplementation and vegetable consumption appear to be associated with lower GrimAgeAccel (41); however, as this association was made from an observational study, further validation from prospective clinical trials is required. Application of epigenetic clock models to epigenomic data from longitudinal studies or dietary interventions to measure biological age and age acceleration offer immense potential for elucidating how dietary interventions can modulate the aging and disease processes.

It also appears that sex and genotype may play a role in modulating epigenetic age acceleration in response to dietary factors. The epigenetic age acceleration lowering of ω-3 PUFAs also appears to be more pronounced in males (GrimAgeAccel: r = −0.08, *P* = 0.012) than in females (r = −0.05, *P* = 0.07). Furthermore, epigenome-wide methylation results from the B-Vitamins for the PRevention Of Osteoporotic Fractures (B-PROOF) study, intervening with daily folic acid and vitamin B-12 supplements in a robust 2-y RCT (73), were inputted into the online DNA methylation age calculator to demonstrate that AgeAccel is reduced in women with the *MTHFR* 677CC but not the 677TT genotype (90). Careful consideration of sex and genotype must therefore be undertaken in the design of epigenetic studies.

In the first and currently the only study to indicate the possibility of reversal of biological age, the Thymus Regeneration, Immunorestoration, and Insulin Mitigation (TRIIM) trial used a cocktail of drugs comprising recombinant human growth hormone (rhGH) to prevent or reverse signs of immunosenescence in a 1-y pilot trial of healthy men aged 51–65 y showing a regression of epigenetic age of −2.5 y on average (70). Although the trial was small (*n* = 9) and, crucially, did not include a control arm, suggestions of biological age reversal were found in all 4 robust methylation clocks available, and in each individual. This study was the first to indicate that potential regression of multiple aspects and biomarkers of aging, including immune function, was possible in humans (70).

Although itself not a dietary factor, it is interesting to note that growth hormone, the supplement chosen in the aforementioned epigenetic age reversal trial, has been noted to perturb mRNA and protein concentrations of DNMT1 (91) and it has been postulated that the age-related dysfunction of growth hormone may play a role in the reduction of DNMTs in aging (82). Further roles for age-related dietary factors such as SAM and α-ketoglutarate (AKG) have been suggested to alter activity of DNMTs and their counterpart TET enzymes during the aging process. The observed age-associated decline in genome-wide methylation may be exacerbated by an observed age-related decline of the essential DNMT substrate, SAM (92, 93), which could result in demethylation of some clock CpGs. Indeed DNMT enzymes also decrease with age in some tissues (91, 94). Furthermore, the hypermethylation of specific loci during aging may be attributable to the decline in AKG and ensuing reductions in TET enzyme activity (82). AKG declines with age (95), reducing its availability as a cofactor for TETs in active demethylation reactions and ensuing hypermethylation of locus-specific regions (96). In support of this theory, AKG has recently been demonstrated to be a rate‐limiting factor controlling DNA demethylation in aging mice (95). This remains speculative, however, because no studies to date have investigated the specific effects of these nutrients on enzyme activity or epigenetic aging.

Despite their obvious strengths, DNA methylation-based clocks are unlikely to replace existing clinical biomarkers and measurements such as blood pressure, walking speed, and grip strength, which are cost-effective and easy to perform. The cost of measuring DNA methylation age prevents the standard adoption of this method, at least until it becomes more affordable. In fact, GrimAge is 61% more accurate than chronological age and 46% more accurate than previously reported epigenetic clocks in predicting time to coronary heart disease. However, despite this significant advancement, neither chronological nor GrimAge are entirely accurate estimators of coronary heart disease and further work is required to determine their role as predictors of cardiovascular and other disease outcomes.

## Methodological Aspects of Studies Investigating DNA Methylation and Diet

Despite the growing interest in the role of diet in influencing DNA methylation and age-related disease, most previous studies in humans were not designed with DNA methylation as the primary outcome, resulting in limited data to provide concrete evidence linking the diet to DNA methylation. The methodological aspects of appropriate study design for the investigation of diet and DNA methylation will be discussed further.

**TABLE 3:** Associations between epigenetic age and age-related conditions

Study	Study design	Population	Sample size (*n*)	Age estimator	Source of DNA	Age-related condition	Association
Cross-sectional studies							
Fiorito et al., 2019 (54)	Cross-sectional	17 cohorts from Europe, the USA, and Australia	16,245	Horvath EAA	Blood	Obesity	Obesity (BMI ≥ 30) associated with higher EAA (β = 0.43, CI: 0.24, 0.61, *P* < 0.001)
Fiorito et al., 2019 (54)	Cross-sectional	17 cohorts from Europe, the USA, and Australia	16,245	Hannum EAA	Blood	Obesity	Obesity (BMI ≥ 30) associated with higher EAA (β = 0.20, CI: 0.05, 0.34, *P* < 0.05)
Fiorito et al., 2019 (54)	Cross-sectional	17 cohorts from Europe, the USA, and Australia	16,245	Levine EAA	Blood	Obesity	Obesity (BMI ≥ 30) associated with higher Levine EAA (β = 1.01 CI: 0.74, 1.28, *P* < 0.001).
Hillary et al., 2019 (97)	Cross-sectional	Lothian Birth Cohort 1936	709	DNAm GrimAge	Whole blood	Cognitive performance	Higher DNAm GrimAge associated with lower cognitive ability (β = −0.18, *P* = 8 x 10^-6^), brain vascular lesions in older age independent of early life cognitive ability
Irvin et al., 2018 (88)	Cross-sectional	Genetics of Lipid Lowering Drugs and diet Network (GOLDN) study	830	Horvath EAA	Blood	Inflammatory markers	EAA marginally associated with increased postprandial HDL (*P* = 0.05), increased postprandial total cholesterol (*P* = 0.06), and decreased soluble IL2 receptor subunit α (*P* = 0.02)
Irvin et al., 2018 (88)	Cross-sectional	Genetics of Lipid Lowering Drugs and diet Network (GOLDN) study	830	Hannum EAA	Blood	Inflammatory markers	EEAA inversely associated with fasting HDL (*P* = 0.02), positively associated with postprandial TG (*P* = 0.02), IL6 (*P* = 0.007), C-reactive protein (*P* = 0.0001), and TNFα (*P* = 0.0001)
Levine et al., 2018 (78)	Cross-sectional	Women's Health Initiative Study (WHI), Framingham Heart Study (FHS), Normative Aging Study (NAS), Jackson Heart Study (JHS)	9164	DNAm PhenoAge	Whole blood	Coronary heart disease	Higher DNAm PhenoAge associated with increased risk of coronary heart disease (β = 0.016–0.073; *P* = 3.35 x 10^-11^).
Levine et al., 2018 (78)	Cross-sectional	Religious Order Study (ROS), Memory and Aging Project (MAP)	700	DNAm PhenoAge	Dorsolateral prefrontal cortex postmortem samples	Alzheimer's disease	DNAm PhenoAge positively associated with neuropathological hallmarks of Alzheimer's disease, such as amyloid load (r = 0.094, *P* = 0.012), neuritic plaques (r = 0.11, *P* = 0.0032), and neurofibrillary tangles (r = 0.10, *P* = 0.0073)
Levine et al., 2018 (78)	Cross-sectional	Women's Health Initiative (WHI) Study	4,177	DNAm PhenoAge	Whole Blood	Blood pressure	Positive association between PhenoAge and systolic BP (r = 0.08, *P* = 1 x 10^-6^)
Lu et al., 2019 (79)	Cross-sectional	Framingham Heart Study (FHS), Women's Health Initiative (WHI) study, the InCHIANTI cohort study, Jackson Heart Study (JHS)	7375	AgeAccelGrim	Whole blood	Time-to-death/coronary heart disease/cancer	AgeAccelGrim strongly associated with time‐to‐death (HR = 1.10, *P* = 2×10‐75), time‐to‐coronary heart disease (HR = 1.07, *P* = 6.2 x 10^‐24^), time‐to‐cancer (HR = 1.07, *P* = 1.3 x 10^‐12^), and hypertension (OR = 1.04, *P* = 5.1 x 10^-13^)
McCrory et al., 2019 (98)	Cross-sectional	The Irish Longitudinal Study on Ageing (TILDA) cohort	490	Horvath EAA	Buffy coat	Allostatic load	Allostatic load not significantly associated with EAA (β = 0.11, CI: −0.16, 0.38, *P* < 0.05)
McCrory et al., 2019 (98)	Cross-sectional	The Irish Longitudinal Study on Ageing (TILDA) cohort	490	Hannum EAA	Buffy coat	Allostatic load	Allostatic load not significantly associated with EAA (β = 0.06, CI: −0.21, 0.33, *P* < 0.05).
McCrory et al., 2019 (98)	Cross-sectional	The Irish Longitudinal Study on Ageing (TILDA) cohort	490	Levine EAA	Buffy coat	Allostatic load	Allostatic load significantly associated with Levine EAA (β = 0.42, CI: 0.24, 0.60, *P* < 0.001)
Quach et al.,2017 (58)	Cross-sectional	Women's Health Initiative study/ InCHIANTI study	4575	EEAA	Whole blood	Blood pressure	EEAA significantly associated with systolic BP (r = 0.07, *P* = 4 x 10^-6^)
Vetter et al., 2019 (99)	Cross-sectional	Berlin Aging Study II	1790	IEAA	Whole blood	Telomere length	rLTL is inversely associated with DNAm age acceleration (β = −0.002, *P* = 0.007)
Case-control studies							
Horvath & Ritz, 2015 (77)	Case-control	The Parkinson's disease, Environment & Genes (PEG) study	592	EEAA	Blood	Parkinson's disease	Parkinson's disease status positively associated with EEAA (*P* = 0.0061)
Horvath & Ritz, 2015 (77)	Case-control	The Parkinson's disease, Environment & Genes (PEG) study	592	Horvath Age Accel	Blood	Parkinson's disease	Parkinson's disease status positively associated with Horvath age acceleration (*P* = 0.06)
Horvath & Ritz,2015 (77)	Case-control	The Parkinson's disease, Environment & Genes (PEG) study	592	IEAA	Blood	Parkinson's disease	Parkinson's disease status positively associated with IEAA (*P* = 0.019)
Perna et al.,2016 (89)	Case-cohort study	ESTHER cohort	1864	Horvath AgeAccel	Whole blood	CVD, cancer	AgeAccel associated with CVD mortality (HR = 1.20; 95% CI: 1.02–1.42), and cancer mortality (HR = 1.20; 95% CI: 1.03–1.39)

BP, blood pressure; CVD, cardiovascular disease; EAA, epigenetic age acceleration; EEAA, extrinsic epigenetic age acceleration; ESTHER, Epidemiologische Studie zu Chancen der Verhütung, Früherkennung und optimierten Therapie chronischer Erkrankungen in der älteren Bevölkerung;; IEAA, intrinsic epigenetic age acceleration; InCHIANTI, Invecchiare in Chianti, aging in the Chianti area; rLTL, relative leukocyte telomere length; TG, triglyceride.

**TABLE 4:** Studies investigating dietary factors and epigenetic age or epigenetic age acceleration

Study	Study design	Population	Dietary factor	Sample size (*n*)	Age estimator	Source of DNA	Effect
Randomized trials and intervention studies							
Chen et al., 2019 (100)	Randomized clinical trial	Overweight/obese African Americans	Vitamin D-3	51	Horvath DNAm age	Buffy coat	Supplementation with 4000 IU/d vitamin D-3 associated with 1.85 y decrease in Horvath epigenetic age compared with placebo (*P* = 0.046)
Chen et al., 2019 (100)	Randomized clinical trial	Overweight/obese African Americans	Vitamin D-3	51	Horvath DNAm age	Buffy coat	Serum 25(OH)D concentrations significantly associated with decreased Horvath ∆Age (*P* = 0.002), independent of treatment
Chen et al., 2019 (100)	Randomized clinical trial	Overweight/obese African Americans	Vitamin D-3	51	Hannum DNAm age	Buffy coat	Supplementation with 2000 IU/d vitamin D-3 associated with 1.90 y decrease in Hannum epigenetic age (*P* = 0.044)
Sae-Lee et al., 2018 (90)	Randomized controlled trial	B-vitamins for the Prevention of Osteoporotic Fractures (B-PROOF) study	Folic acid, vitamin B-12	44	Horvath Age Accel	Buffy coat	Reduced age acceleration in response to folic acid and vitamin B-12 supplementation in women with *MTHFR* 677CC genotype (*P* = 0.04)
Sae-Lee et al., 2018 (90)	Intervention study	Nonobese healthy male smokers	Monomeric and oligomeric flavanol	13	Horvath Age Accel	Leukocytes	No change in age acceleration in response to monomeric and oligomeric flavanol (MOF) supplementation
Cross-sectional studies							
Levine et al., 2018 (78)	Cross-sectional	Women's Health Initiative (WHI) study	Carotenoids	2267	PhenoAge Accel	Whole blood	Lower PhenoAgeAccel associated with increased mean intake of carotenoids (r = –0.22, *P* = 2 x 10^-27^), lycopene (r = –0.11, *P* = 3 x 10^-3^), α-carotene (r = –0.19, *P* = 5 x 10^-20^), β-carotene (r = –0.18, *P* = 2 x 10^-17^), lutein + zeaxanthin (r = –0.17, *P* = 2 x 10^-16^), β-cryptoxanthin (r = –0.17, *P* = 2 x 10^-15^) but positively associated with γ-tocopherol (r = 0.07, *P* = 6 x 10^-4^)
Lu et al., 2019 (79)	Cross-sectional	Framingham Heart Study (FHS)	ω-3 PUFAs	2174	AgeAccelGrim	Whole blood	ω-3 PUFAs and vegetable intake associated with lower GrimAge (r = –0.10, *P* = 4.6 x 10^-7^, linear mixed effects *P* = 1.3 x 10^-5^). Effect more pronounced in males (r = –0.08, *P* = 0.012) than in females (r = –0.05, *P* = 0.07)
Quach et al., 2017 (58)	Cross-sectional	Women's Health Initiative study/InCHIANTI study	Carotenoids	4575	EEAA	Whole blood	Lower EEAA significantly associated with higher mean plasma carotenoid concentrations (r = –0.13, *P* = 2 x 10^-9^), α-carotene (r = –0.11, *P* = 9 x 10^-8^), β-carotene (r = –0.11, *P* = 3 x 10^-7^), lutein + zeaxanthin (r = –0.9, *P* = 1 x 10^-5^), β-cryptoxanthin (r = –0.11, *P* = 3 x 10^-7^), and lower γ-tocopherol (r = 0.09, *P* = 9 x 10^-6^)
Quach et al., 2017 (58)	Cross-sectional	Women's health Initiative/InCHIANTI study	Fish	4575	EEAA	Whole blood	Lower EEAA associated with higher intake of fish (t_meta_ = –2.92, p_meta_ = 0.003)
Quach et al., 2017 (58)	Cross-sectional	Women's Health Initiative study/ InCHIANTI study	Tocopherol	4575	IEAA	Whole blood	Lower IEAA associated with lower plasma γ-tocopherol (r = 0.08, *P* = 2 x 10^-4^)

EEAA, extrinsic epigenetic age, IEAA, intrinsic epigenetic age; InCHIANTI, Invecchiare in Chianti, aging in the Chianti area; MTHFR, methyleneterahydrofolate reductase; p_meta_, p-valu e meta-analysis; t_meta_, t-value meta-analysis; 25(OH)D, 25-hydroxyvitamin D.

### Study design and population

The study design utilized as well as dietary or biochemical data collected are critical when investigating the link between nutrient intake or status and DNA methylation. The majority of studies so far are observational and have provided inconsistent evidence for the role of dietary factors, especially B-vitamins, in modulating DNA methylation, perhaps owing to inconsistencies in study design and choice of assay (101). Although observational studies offer the advantage of providing comprehensive data with large sample sizes and highlight associations between nutrients and DNA methylation, they are unable to provide clarity with respect to dietary causality. RCTs represent a robust study design for establishing the effects of B-vitamins on DNA methylation; however, studies of this nature are lacking. Although no study on its own can prove causality, randomization in RCTs reduces bias and provides a rigorous tool to examine cause-effect relations between an intervention and an outcome (102). Additionally, apart from establishing the biological roles of B-vitamins in modulating DNA methylation, there is a need for RCTs to further incorporate dose-response design in order to determine the optimum doses of B-vitamins required to modulate DNA methylation. Longitudinal studies that assess methylation in individuals at several time points, and thereby reduce noise in the methylation signal owing to interindividual variation, are particularly useful in helping to elucidate the role of diet and methylation in disease. Furthermore, the majority of existing studies have employed FFQs in estimating dietary intake, yielding only semiquantitative data, prone to measurement errors that may not accurately reflect status, resulting in misclassification which can compromise the ability to detect statistically significant associations (103). Importantly, biochemical biomarker concentrations of status provide more reliable indicators than dietary intake to investigate the relation between B-vitamins and DNA methylation.

### Novel approaches for DNA methylation analyses in nutrition studies

Methods to examine DNA methylation have evolved over the years and have become more sophisticated. Although commonly used methods including HPLC-UV, LC-MS/MS, methyl acceptance assay, and pyrosequencing are still useful in analyses of DNA methylation, novel technologies such as the Infinium HumanMethylation450K BeadChip array (450K) or the Infinium MethylationEPIC BeadChip (850K) microarray provide higher resolution for analyzing DNA methylation on a genome-wide scale (104, 105). Although not offering as much genome coverage as whole-genome bisulfite sequencing (WGBS), the Illumina arrays analyze a significant proportion of total sites for DNA methylation at 853,307 CpG sites (EPIC/850K) and 485,764 CpG sites (450K) across the human genome. The CpG sites interrogated by the 850K array include 439,562 CpGs out of 482,421 CpGs included in the 450K microarray and an additional 413,745 new CpG sites that were not included in the 450K microarray. The EPIC array provides a highly reliable genomic platform for studying DNA methylation patterns across the genome especially in underexplored territories including enhancer sequences (106). Furthermore, in comparison to WGBS, Illumina microarrays provide good value for money in terms of desired coverage, resolution, and number of samples that can be analyzed, providing large amounts of high-quality data that can be easily input into epigenetic clock algorithms.

Advantages of using these approaches include the production of large datasets that can be analyzed by streamlined analytical pipelines, providing important information on the epigenome-wide landscape. Several sophisticated computational tools and software are available for the analysis and interpretation of large EWAS datasets. The relevant concepts, computational methods, and software for the analysis and interpretation of large DNA methylation data as well as statistical considerations have been thoroughly reviewed by Bock and colleagues (107, 108). These statistical approaches allow for computation of epigenetic age, and are able to control for false discovery rates and adjust for cell and tissue variation, which are all major sources of confounding in DNA methylation studies. Some of the popular and widely used software for processing and analysis of bisulfite microarray data in particular include *minfi* (109), RnBeads (110), The Chip Analysis Methylation Pipeline (ChAMP) (111), and methylumi (112). Furthermore, other software packages such as dmrFinder (113), DMRcate (14), and IMA (114) are available for the identification of differentially methylated regions (DMRs). New platforms such as CandiMeth (https://github.com/sjthursby/CandiMeth) are also making it easier for those with little bioinformatics experience to look at methylation across the genome in samples for which array data is available.

## Conclusion

Nutritional epigenomics has highlighted diet as a critical factor with the potential to influence both healthspan and lifespan. Novel insights into how perturbations in 1-carbon metabolism influence DNA methylation and data from epigenome-wide studies of nutrition interventions offer promising insights to understanding how diet impacts the methylome during healthy aging and disease. Epigenetic clocks provide an exciting additional insight into how preventive and treatment strategies may increase the healthspan of an aging global population. Despite the heightened research interests in nutritional epigenomics, the field is still beset with several methodological challenges, which greatly impact the quality of evidence currently available. The population under study must be extensively characterized to identify and exclude possible confounding factors. Robust study designs, which utilize randomization and measure appropriate biomarkers, are required to clarify the factors underlying epigenetic aging. Replication and validation of findings in multiple independent cohorts are essential to reduce reporting of false positive findings. Epigenetic clocks described here have sampled individuals from a wide spectrum of ages. A DNA methylation clock which focuses on older people or those with specific diseases could help to more accurately predict age-related disease and help to identify factors which delay or prevent this progression. Improvements in estimating time to disease have been made in the latest GrimAge clock, which is significantly more predictive than chronological age in estimating time to various diseases; however, much additional research is required to advance our knowledge and understanding in relation to coronary heart disease. Longitudinal studies offer the important advantage of tracking individuals over extended periods to enable the identification of factors which influence the diagnosis and treatment of disease, making these studies particularly valuable for clarifying whether observed changes in DNA methylation are a result of disease or have a causal role. A better understanding of the DNA methylome during aging will offer the opportunity to promote healthy aging and identify nutritional interventions which delay or prevent age-related disease in order to influence public health outcomes and policies.

## Notes

The research described in this review was supported in part by Northern Ireland Chest Heart & Stroke Association (NICHS206_07; DJL-M and MW), DSM Nutritional Products (MW, HN, JJS, and CH), Economic and Social Research Council/Biotechnology and Biological Sciences Research Council (ES/N000323/1; CPW and DJL-M), and Vice Chancellor's Research Scholarship, Ulster University (SDA). SDA was the recipient of travel grants from the Nutrition Society, Biochemical Society, and Genetics Society.

None of the entities providing support were involved in the design, implementation, analysis, interpretation of the data, or in the production of the final review manuscript.

Author disclosures: MW, HN, and JJS hold an international patent on the use of riboflavin in the treatment of blood pressure. All other authors report no conflicts of interest.

Abbreviations used: CGI, CpG island; CVD, cardiovascular disease; DMR, differentially methylated region; DNMT, DNA methyltransferase; EEAA, extrinsic epigenetic age; EWAS, epigenome-wide association studies; IEAA, intrinsic epigenetic age; MTHFR, methylenetetrahydrofolate reductase; MZ, monozygotic; RCT, randomized controlled trial; SAM, S-adenosylmethionine; TET, ten-eleven translocation methylcytosine dioxygenase enzyme; WGBS, whole-genome bisulfite sequencing; 5mC, 5-methyl cytosine.
